# Cost-effectiveness of sotorasib as a second-line treatment for non-small cell lung cancer with KRASG^12C^ mutation in China and the United States

**DOI:** 10.3389/fphar.2024.1348688

**Published:** 2024-06-14

**Authors:** Ya-Ning Zhu, Meng Tang, Ke-Xin Sun, Bei Gao, Xian-Peng Shi, Peng Zhang

**Affiliations:** ^1^ Department of Pharmacy, Shaanxi Provincial People’s Hospital, Xi’an, China; ^2^ Department of Pharmacy, Xijing Hospital, Fourth Military Medical University, Xi’an, China; ^3^ Department of Pharmacy, Gansu Provincial Hospital, Lanzhou, China

**Keywords:** cost-effectiveness, NSCLC, KRASG^12C^ mutation, sotorasib, China

## Abstract

**Purpose:**

To evaluate the cost-effectiveness of sotorasib versus docetaxel in non-small cell lung cancer (NSCLC) patients with KRAS^G12C^ mutation from the China and United States’social perspective.

**Materials and Methods:**

A Markov model that included three states (progression-free survival, post-progression survival, and death) was developed. Incremental cost-effectiveness ratio (ICER), quality-adjusted life-year (QALY), and incremental QALY were calculated for the two treatment strategies. One-way sensitivity analysis was used to investigate the factors that had a greater impact on the model results, and tornado diagrams were used to present the results. Probabilistic sensitivity analysis was performed with 1,000 Monte Carlo simulations. Assume distributions based on parameter types and randomly sample all parameter distributions each time., The results were presented as cost-effectiveness acceptable curves.

**Results:**

This economic evaluation of data from the CodeBreak 200 randomized clinical trial. In China, sotorasib generated 0.44 QAYL with a total cost of $84372.59. Compared with docetaxel, the ICER value of sotorasib was $102701.84/QALY, which was higher than willingness to pay (WTP), so sotorasib had no economic advantage. In the US, sotorasib obtained 0.35 QALY more than docetaxel, ICER was $15,976.50/QALY, which was more than 1 WTP but less than 3 WTP, indicating that the increased cost of sotorasib was acceptable. One-way sensitivity analysis showed that the probability of sotorasib having economic benefits gradually increased when the cost of follow-up examination was reduced in China. And there was no influence on the conclusions within the range of changes in China. When the willingness to pay (WTP) exceeds $102,500, the probability of sotorasib having cost effect increases from 0% to 49%.

**Conclusion:**

Sotorasib had a cost effect from the perspective in the United States. However, sotorasib had no cost effect from the perspective in China, and only when the WTP exceeds $102,500, the probability of sotorasib having cost effect increases from 0% to 49%.

## Introduction

Lung cancer is the leading cause of cancer death worldwide, with an estimated 1.8 million deaths, accounting for 18.7% of all cancer deaths (Bray et al., 2024). Lung cancer was the cancer with the highest incidence and mortality rate in China, with 733,300 deaths in 2022 (Zheng et al., 2024). Non-small cell lung cancer (NSCLC) is the most common type of lung cancer, accounting for about 80%–85% (Zhou et al., 2017). In China, approximately 70% of patients are diagnosed with advanced stages and do not have the opportunity to undergo surgery (Zhou et al., 2017). In the past 10 years, the identification of specific driver mutant genes and the development of targeted therapy have changed the pattern of treatment of advanced NSCLC patients. KRAS gene mutation is the most common driver gene in NSCLC (Aredo et al., 2019). A study in western countries reported that 27% of lung adenocarcinoma patients carry KRAS mutations, with KRAS^G12C^ mutations reaching as high as 39% (El Osta et al., 2019). Meanwhile, a study reported that 10% of NSCLC patientshave KRAS mutations, and nearly 30% of them develop KRAS^G12C^ mutations in China (Liu et al., 2020). Due to the special structural type of KRAS, it had long been considered an undruggable target (Cox et al., 2014). In the past, the main treatment option for patients with KRAS mutations was chemotherapy, but previous studies have shown that chemotherapy had little effect on patients with KRAS mutations (Hames et al., 2016). Until the emergence of sotorasib broke the deadlock of KRAS mutation without targeted drugs.

Sotorasib is the first KRAS^G12C^ inhibitor successfully developed in the world after more than 40 years of KRAS mutant protein research. Its main mechanism of action is to irreversibly inhibit the small molecules of the KRAS^G12C^ target, block KRAS signal, inhibit cell growth, and promote cell apoptosis. In 2021, sotorasib was approved by the Food and Drug Administration (FDA), becoming the world’s first drug specifically for the treatment of locally advanced or metastatic NSCLC with KRAS^G12C^ mutation (Blair, 2021). In a single-arm, Phase II trial that included 124 patients with advanced NSCLC with KRAS^G12C^ mutation who had previously received standard were and received oral sotorasib (960 mg, qd), 100 (79.3%) patients developed disease control, complete response occurred in four patients (3.2%) and partial response occurred in 42 patients (33.9%) (Skoulidis et al., 2021). A Phase III study (de et al., 2023) showed that the median progression-free survival (PFS) of sotorasib and docetaxel was 5.6 months and 4.5 months, respectively. The 12-month PFS were 24.8% and 10.1%, respectively. Based on these clinical trial results, National Comprehensive Cancer Network (NCCN)guidelines and Chinese Society of Clinical Oncology (CSCO) guidelines recommend sotorasib as a second-line treatment for NSCLC with KRAS^G12C^ mutations (NCCN Clinical Practice Guidelines in Oncology NCCN Guidelines^®^, 2022; Organized by the Guideline Working Committee of the Chinese Society of Clinical Oncology, 2023).

Sotorasib undoubtedly provides a longer chance of survival for patients with KRAS^G12C^ NSCLC. However, sotorasib is costly and can increase the financial burden on patients. Pharmacoeconomics compares different drug administration regimens through cost analysis to select the most cost-effective regimen for patients (Schulman and Linas, 1997). There is no economic evaluation of sotorasib for the treatment of KRAS^G12C^ mutated NSCLC.

Therefore, this study evaluated the cost-effectiveness of sotorasib versus chemotherapy in patients with KRAS^G12C^ mutation NSCLC to support clinical and patient medication selection from the perspective of China and the United States (US).

## Materials and methods

### Model structure

**FIGURE 1:**
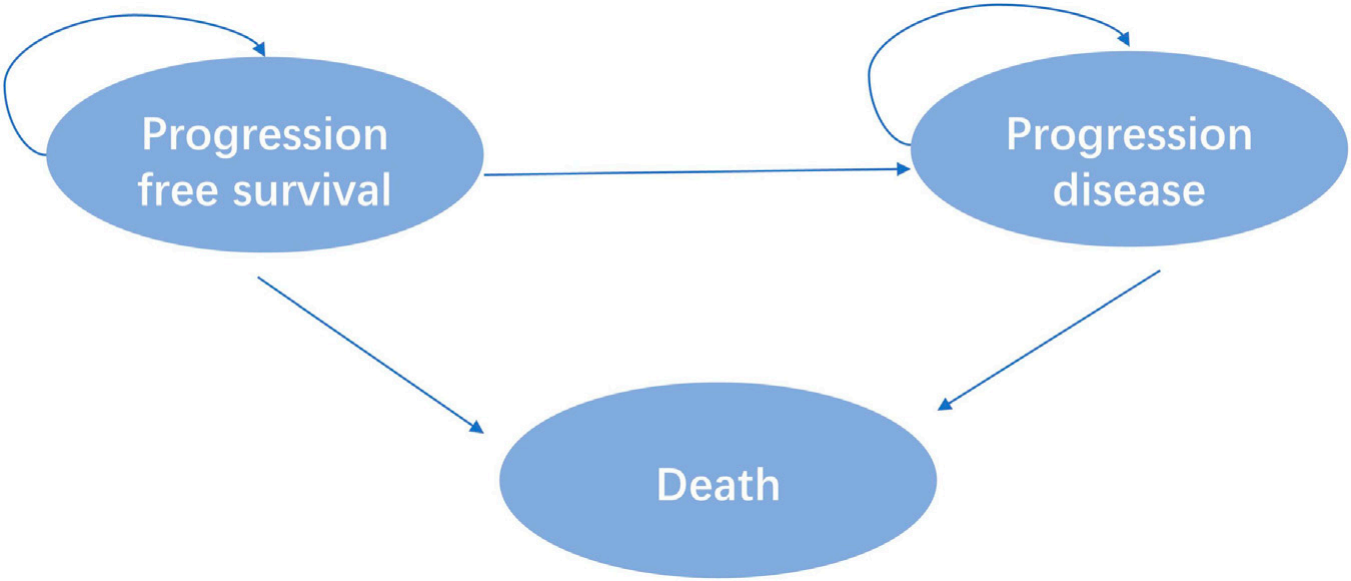
Model structure.

Eligible patients included were at least 18 years of age with advanced NSCLC with disease progression with a KRAS^G12C^ mutation following prior treatment with platinum-based chemotherapy and Programmed cell death protein 1 (PD-1) or Programmed cell death one ligand 1(PD-L1) inhibitors in the CodeBreak 200 clinic trial. A total of 345 patients were randomly assigned 1:1 to sotorasib or docetaxel. According to the outcome of advanced NSCLC disease, combined with the relevant literature of published clinical trials and economic evaluation (Cai et al., 2019). This study divided the disease model of advanced NSCLC into three independent and mutually exclusive health states: progression-free survival (PFS), progressive disease (PD) and death, as shown in Figure 1.

### Model parameter

The PFS and OS curves of sotorasib and docetaxel were based on the results of the clinical trial (NCT04303780). We collected data points from PFS and OS curves by GetData Graph Digitizer (version 2.26), these data points were used to fit the following survival function, by using Exponential, Weibull, Gamma, Gengamma, Gompertz, Lognormal, and Loglogistic distributions to fit the data. According to the Akaike Information Criterion (AIC), R software was used to calculate the AIC value of each distribution, and the smallest distribution of AIC was the best fitting method for PFS and OS curves (Supplementary Figures S1, S2), and the parameter mean μ and standard variance σ were obtained. Our results showed that the lognormal model was the most reasonable function for extrapolating OS and PFS from the sotorasib and docetaxel groups. The key clinical parameter inputs were listed in Table 1 and Supplementary Table S1.

**TABLE 1:** Parameters input to the model.

		Chinese perspective					
Items	Drug	Variable nam	Parameter values	Upper limit	Lower limit	Distribution	Reference/source
Cost of medications ($)		C_Sotorasib	16,892.82	20,271.384	13,514.256	Gamma1	Micro medex redbook (IBM Micromedex, 2020)
		C_docetaxel	228.41	2,556.05	11.83	Gamma1	Micro medex redbook (IBM Micromedex, 2020)
		U_PFS	0.804	0.84	0.536	Beta	Nafees B, et al. (2017) (Nafees et al., 2017)
		U_PD	0.321	0.321	0.031	Beta	Nafees B, et al. (2017) (Nafees et al., 2017)
Utility value of the adverse reaction	Sotorasib	U_Diarrhea_S	0.22	0.26	0.18	Beta	Nafees B, et al. (2017) (Nafees et al., 2017)
		U_Fatigue_S	0.29	0.35	0.23	Beta	Nafees B, etall. (2017) (Nafees et al., 2017)
		U_Nausea_S	0.02	0.24	0.16	Beta	Nafees B, et al. (2017) (Nafees et al., 2017)
		U_Anemia_S	0.07	0.09	0.06	Beta	Nafees B, et al. (2008) (Nafees et al., 2008)
		U_Decreasedappetite_S	0.39	0.47	0.12	Beta	Lemmon CA, et al. (2022) (Lemmon et al., 2022)
	Docetaxel	U_Diarrhea_D	0.22	0.26	0.18	Beta	Nafees B, et al. (2017) (Nafees et al., 2017)
		U_Fatigue_D	0.29	0.35	0.23	Beta	Nafees B, et al. (2017) (Nafees et al., 2017)
		U_Nausea_D	0.20	0.24	0.16	Beta	Nafees B, et al. (2017) (Nafees et al., 2017)
		U_Anaemia_D	0.07	0.09	0.06	Beta	Nafees B, et al. (2008) (Nafees et al., 2008)
		U_Neutropenia_D	0.35	0.42	0.28	Beta	Nafees B, et al. (2017) (Nafees et al., 2017)
		U_Mucositis_D	0.53	0.57	0.47	Beta	Soto-Perez-de-Celis E, et al. (2018) (Soto-Perez et al., 2019)
		U_Febrile neutropenia_D	0.47	0.56	0.38	Beta	Nafees B, et al. (2017) (Nafees et al., 2017)
		U_Pneumonia_D	0.50	0.60	0.40	Beta	Lemmon CA, et al. (2022) (Lemmon et al., 2022)
Cost of adverse reactions ($)	Sotorasib	C_Diarrhoea_S	1,209.58	1,451.50	967.66	Beta	Medicwere (Centers for Medicare and Medicaid Services, 2024)
		C_Fatigue_S	1,185.92	1,423.10	948.74	Beta	Medicwere (Centers for Medicare and Medicaid Services, 2024)
		C_Nausea_S	1,209.58	1,451.50	967.66	Beta	Medicwere (Centers for Medicare and Medicaid Services, 2024)
		C_Anaemia_S	5,394.51	6,473.41	4,315.61	Beta	Medicwere (Centers for Medicare and Medicaid Services, 2024)
		C_Decreasedappetite_S	1,209.58	1,451.50	967.66	Beta	Medicwere (Centers for Medicare and Medicaid Services, 2024)
	Docetaxel	C_Diarrhoea_D	1,209.58	1,451.50	967.66	Beta	Medicwere (Centers for Medicare and Medicaid Services, 2024)
		C_Fatigue_D	1,185.92	1,423.10	948.74	Beta	Medicwere (Centers for Medicare and Medicaid Services, 2024)
		C_Nausea_D	1,209.58	1,451.50	967.66	Beta	Medicwere (Centers for Medicare and Medicaid Services, 2024)
		C_Anaemia_D	5,394.51	6,473.41	4,315.61	Beta	Medicwere (Centers for Medicare and Medicaid Services, 2024)
		C_Stomatitis_D	1,638.38	1966.06	1,310.70	Beta	Medicwere (Centers for Medicare and Medicaid Services, 2024)
		C_Asthenia_D	1,185.92	1,423.10	948.74	Beta	Medicwere (Centers for Medicare and Medicaid Services, 2024)
		C_Neutropenia_D	2,449.99	2,939.99	1959.99	Beta	Medicwere (Centers for Medicare and Medicaid Services, 2024)
		C_Neuropathyperipheral_D	1,453.53	1744.24	1,162.82	Beta	Medicwere (Centers for Medicare and Medicaid Services, 2024)
		C_Myalgia_D	1932.79	2,319.35	1,546.23	Beta	Medicwere (Centers for Medicare and Medicaid Services, 2024)
		C_Arthralgia_D	1932.79	2,319.35	1,546.23	Beta	Medicwere (Centers for Medicare and Medicaid Services, 2024)
		C_Febrileneutropenia_D	2,449.99	2,939.99	1959.99	Beta	Medicwere (Centers for Medicare and Medicaid Services, 2024)
		C_Pneumonia_D	1789.55	2,147.46	1,431.64	Beta	Medicwere (Centers for Medicare and Medicaid Services, 2024)
The cost of best supportive ($)		C_bestsupportive	4,221	5,065	3,377	Beta	ChengS, et al. (2021) (Cheng et al., 2021)
The cost of terminal care ($)		C_terminal	17,185	20,622	13,748	Beta	Cheng S, et al. (2021) (Cheng et al., 2021)

Items		Chinese perspective					
		Variable Name	Parameter Values	Upper limit	Lower limit	Distribution	Reference/source
Cost of medications ($)		C_Sotorasib	272.19	326.63	217.76	Gamma1	Boao Super Hospital*
		C_docetaxel	148.05	1,299.60	19.42	Gamma1	Yaozhi Net (Yaozhi Net, 2023)
		U_PFS	0.804	0.84	0.536	Beta	Nafees B, et al. (2017) (Nafees et al., 2017)
		U_PD	0.321	0.321	0.031	Beta	Nafees B, et al. (2017) (Nafees et al., 2017)
Utility value of the adverse reaction	Sotorasib	U_Diarrhoea_S	0.07	0.09	0.06	Beta	Nafees B, et al. (2017) (Nafees et al., 2017)
		U_Fatigue_S	0.07	0.09	0.06	Beta	Nafees B, et al. (2017) (Nafees et al., 2017)
		U_Nausea_S	0.12	0.09	0.06	Beta	Nafees B, et al. (2017) (Nafees et al., 2017)
		U_Anaemia_S	0.07	0.09	0.060	Beta	Wan X, et al. (2019) (Wan et al., 2019)
		U_Decreased appetite_S	0.030	0.04	0.02	Beta	Huang X, et al. (2023) (Huang et al., 2023a)
		U_Asthenia_S	0.070	0.09	0.06	Beta	Wan X, et al. (2019) (Wan et al., 2019)
		U_ALT_S	0.050	0.06	0.04	Beta	Huang X, et al. (2023) (Huang et al., 2023a)
		U_AST_S	0.050	0.06	0.04	Beta	Huang X, et al. (2023) (Huang et al., 2023a)
	Docetaxel	U_Diarrhoea_D	0.070	0.09	0.06	Beta	Nafees B, et al. (2017) (Nafees et al., 2017)
		U_Fatigue_D	0.070	0.09	0.06	Beta	Nafees B, et al. (2017) (Nafees et al., 2017)
		U_Nausea_D	0.120	0.090	0.060	Beta	Nafees B, et al. (2017) (Nafees et al., 2017)
		U_Anaemia_D	0.070	0.090	0.060	Beta	Wan X, et al. (2019) (Wan et al., 2019)
		U_Asthenia_D	0.070	0.090	0.060	Beta	Wan X, et al. (2019) (Wan et al., 2019)
		U_Neutropenia_D	0.420	0.504	0.336	Beta	Nafees B, et al. (2017) (Nafees et al., 2017)
		U_Neuropathy peripheral_D	0.180	0.198	0.162	Beta	Weng X, et al. (2020) (Weng et al., 2020)
		U_Myalgia_D	0.070	0.090	0.050	Beta	Huang X, et al. (2023) (Huang et al., 2023b)
		U_Arthralgia_D	0.180	0.198	0.162	Beta	Huang X, et al. (2023) (Huang et al., 2023b)
		U_Febrile neutropenia_D	0.420	0.504	0.336	Beta	Nafees B, et al. (2017) (Nafees et al., 2017)
		U_Pneumonia_D	0.050	0.06	0.04	Beta	Shao T, et al. (2022) (Shao et al., 2022)
Cost of adverse reactions ($)		C_Diarrhoea	5.9	7.08	4.72	Beta	Cai Y, et al. (2021) (Cai et al., 2021)
		C_Fatigue	135.51	162.612	108.408	Beta	Cai Y, et al. (2021) (Cai et al., 2021)
		C_Nausea	14.62	17.544	11.696	Beta	Cai Y, et al. (2021) (Cai et al., 2021)
		C_Anaemia	604.03	724.836	483.224	Beta	Cai Y, et al. (2021) (Cai et al., 2021)
		C_Decreased appetite	115	138	92	Beta	Yang F, et al. (2021) (Yang et al., 2021)
		C_Stomatitis	4.66	5.592	3.728	Beta	Shu Y, et al. (2021) (Shu et al., 2021)
		C_Asthenia	135.51	162.612	108.408	Beta	Cai Y, et al. (2021) (Cai et al., 2021)
		C_Alanine aminotransferase increased	91.9	110.28	73.52	Beta	Zhang X, et al. (2022) (Zhang et al., 2022)
		C_Aspartate aminotransferase increased	91.9	110.28	73.52	Beta	Zhang X, et al. (2022) (Zhang et al., 2022)
		C_Neutropenia	541.85	650.22	433.48	Beta	Cai Y, et al. (2021) (Cai et al., 2021)
		C_Neuropathy peripheral	56.81	68.172	45.448	Beta	Cui J, et al. (2021) (Cui et al., 2021)
		C_Myalgia	136.36	163.632	109.088	Beta	Huang X, et al. (2023) (Huang et al., 2023b)
		C_Vomiting	14.62	17.544	11.696	Beta	Cai Y, et al. (2021) (Cai et al., 2021)
		C_Arthralgia	151.5	181.8	121.2	Beta	Huang X, et al. (2023) (Huang et al., 2023b)
		C_Febrile neutropenia	330	412.5	247.5	Beta	Wang Y, et al. (2022) (Wang et al., 2022)
		C_Pyrexia	845.61	1,056.98	634.21	Beta	Chen P, et al. (2022) (Chen et al., 2022)
		C_Pneumonia	1,640	1968	1,312	Beta	Zhao M, et al. (2023) (Zhao et al., 2023)
The cost of palliative ($)		C_palliative	2,464.5	3,696.75	1971.6	Beta	Cheng S, et al. (2021) (Cheng et al., 2021)
The cost of terminal care ($)		C_terminal	2,205	2,646	1764	Beta	Cheng S, et al. (2021) (Cheng et al., 2021)

### Utility

Health Utility refers to a patient’s preference for a specific health condition or treatment. Patients’ satisfaction with a state of health was assessed on an interval scale of 0–1, where 0 represented death and one represented complete health. Due to the lack of quality of life utility studies in patients with advanced NSCLC in China and the US, the utility values of PFS and PD states included were obtained from an international study of quality of life in patients with advanced NSCLC in this study (Nafees et al., 2017). The utility value of the PFS state was 0.804 (0.536–0.84) and the utility value of the PD state was 0.321 (0.031–0.321) in China. The utility value for the PFS state was 0.754 (0.536–0.84) and the utility value for the PD state was 0.095 (0.031–0.321) in the US.

### Cost

Direct medical costs included the costs of drugs, management of adverse events (AEs), hospitalization cost, routine follow-up, best supportive, palliative and hospice. These costs were estimated from the published literature or institution. Dosage of docetaxel was calculated by body surface area 1.72 m^2^ for Chinese and 1.82 m^2^ for Americans, and the actual single dose was calculated by 140 mg. The incidence of AE was mainly from the clinical trial (NCT04303780) report in this study, mainly considering ≥3AEs, and the cost was mainly from previously published studies and professional websites. All cost values in China are based on the January 2023 exchange rate (1 USD = 6.92 RMB). The costs of adverse drug reactions, hospitalization costs, optimal support costs, palliative care costs, and hospice costs were derived from previously published literature, as shown in Table 1. The prices of drugs were also detailed in Table 1.

### Effectiveness

Cost, QALY, and ICERs were the main output evaluation results. In this study, WTP threshold was set at 1-3 time the *per capita* gross domestic product (GDP) in 2022, which is $12,374.81-37,124.42 in China and $76348-229044 in the US.

### Sensitivity analysis

In pharmacoeconomic evaluation, the result of cost-effectiveness is often biased due to the uncertainty of unreasonable data collection and evaluation methods. Therefore, sensitivity analysis is used to change the test conditions or change the parameter values within a certain range to evaluate its stability to the conclusion (Guanshen et al., 2015). One-way sensitivity analysis and probabilistic sensitivity analysis (PSA) were used to reflect the effects of uncertainty. The range and distribution of parameter variations were shown in Table 1. In the absence of upper and lower limit values, they were calculated as ±20% of the parameters. A second-order Monte Carlo simulation was used to sample 1,000 random simulations and the results are expressed as cost-effectiveness acceptable curves.

## Results

### Analysis of cost-effectiveness

The results of running the model for 120 cycles were shown in Table 2. In China, sotorasib generated 0.35 QAYL with a total cost of $84372.59. Compared with docetaxel, sotorasib increases the effectiveness by 0.09 QALY. ICER was $102701.84, which was higher than 3 times GDP, so sotorasib had no economic advantage. In the US, sotorasib obtained 0.35 QALYsmore than docetaxel, and ICER was $15,976.50/QALY, which was between 1 and 3 times GDP, indicating that the increased cost of sotorasib was acceptable.

**TABLE 2:** Cost-effectiveness analysis of Sotorasib and docetaxel taxel.

Results	China perspective		US perspective	
	Sotorasib	Docetaxel	Sotorasib	Docetaxel
Cost	84,372.59	74,809.61	487,585.57	363,794.84
Incr Cost	9,562.972	-	123,790.73	-
QALYs	0.44	0.35	0.35	−7.39
Incr QALY	0.09	-	7.75	-
ICER	102,701.835	-	15,976.50	-

### One-way sensitivity analysis

**FIGURE 2:**
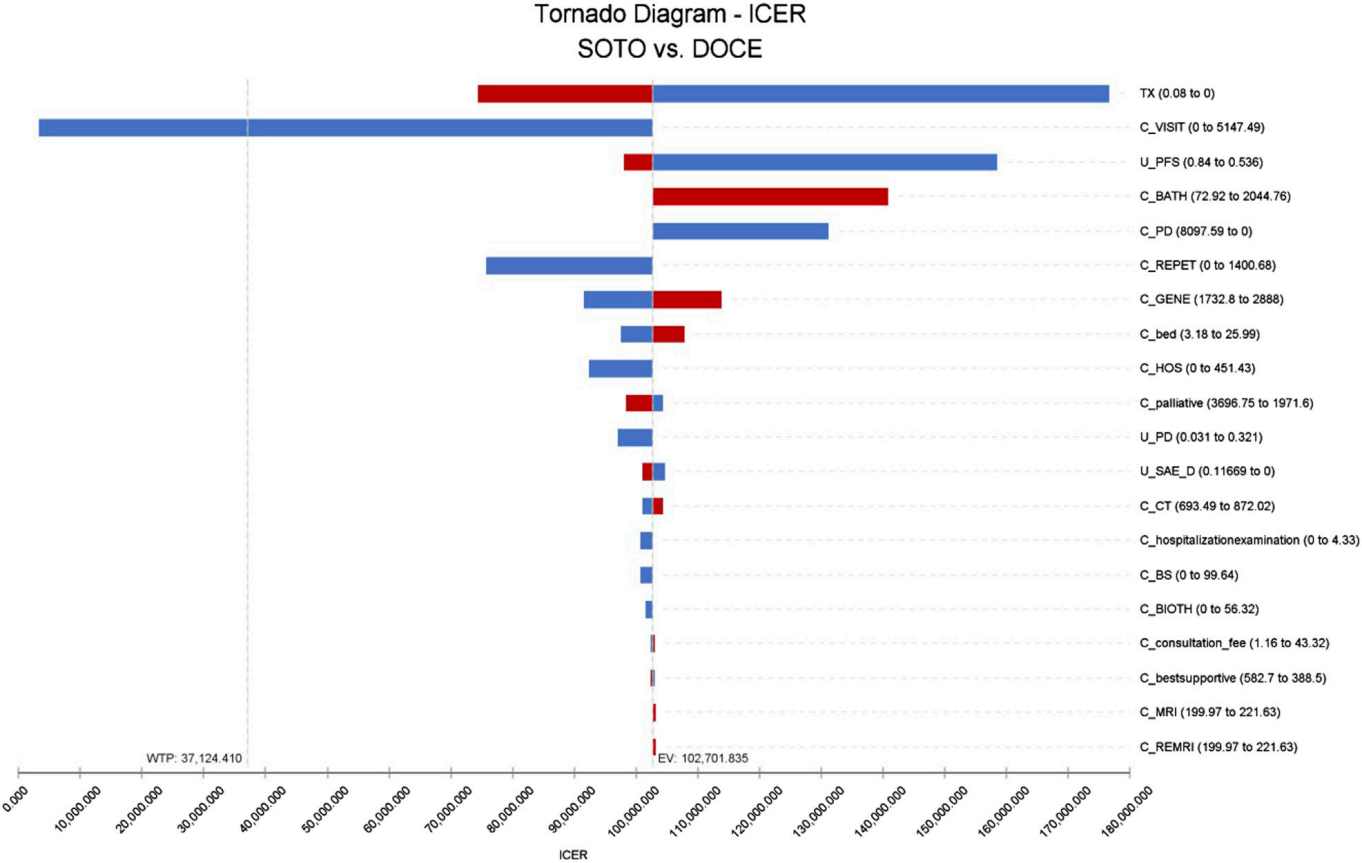
Tornado diagram of sotorasib vs. docetaxel in China perspective. Note: SOTO, sotorasib; DOCE, docetaxel.

The results of one-way sensitivity analysis showed that the three factors that have the greatest influence on the incremental cost-effectiveness ratio was the discount rate, follow-up cost, and the utility value of PFS, as shown in Figure 2. According to the monistic sensitivity analysis (Supplementary Figure S3), when the cost of follow-up examination was reduced, the probability of sotorasib having economic benefits gradually increased. When other parameters vary within the upper and lower limits, ICER had no effect.

**FIGURE 3:**
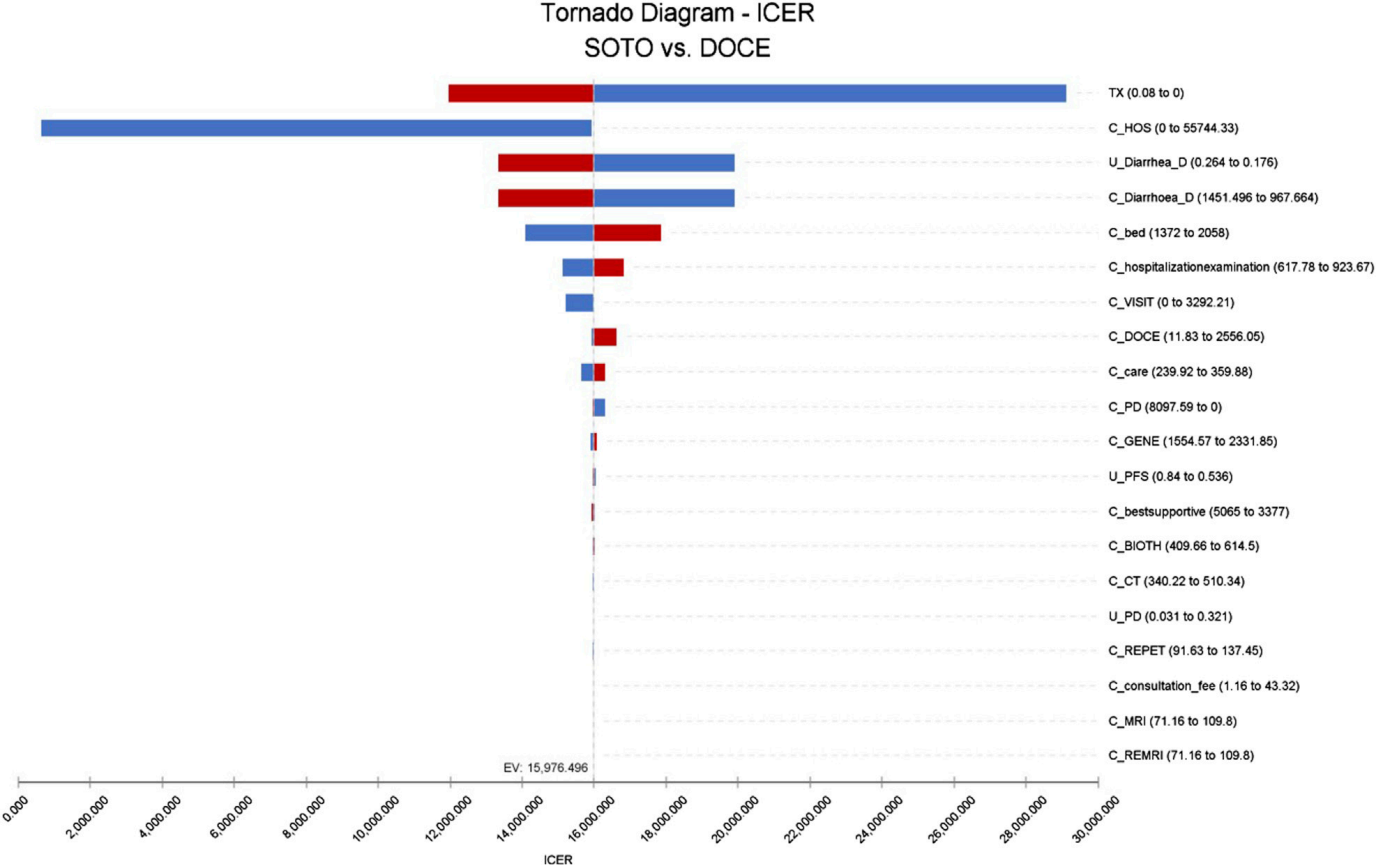
Tornado diagram of sotorasib vs. docetaxel in the US perspective. Note: SOTO, sotorasib; DOCE, docetaxel.

The results of single factor sensitivity analysis showed that the three factors that had the greatest influence on the incremental cost-effectiveness ratio was discount rate, hospitalization cost, and the cost of treating diarrhea with docetaxel. Within the range of changes in the upper-lower limits of the set parameters, there was no influence on the conclusions, as shown in Figure 3.

### Cost-effectiveness acceptability curve

**FIGURE 4:**
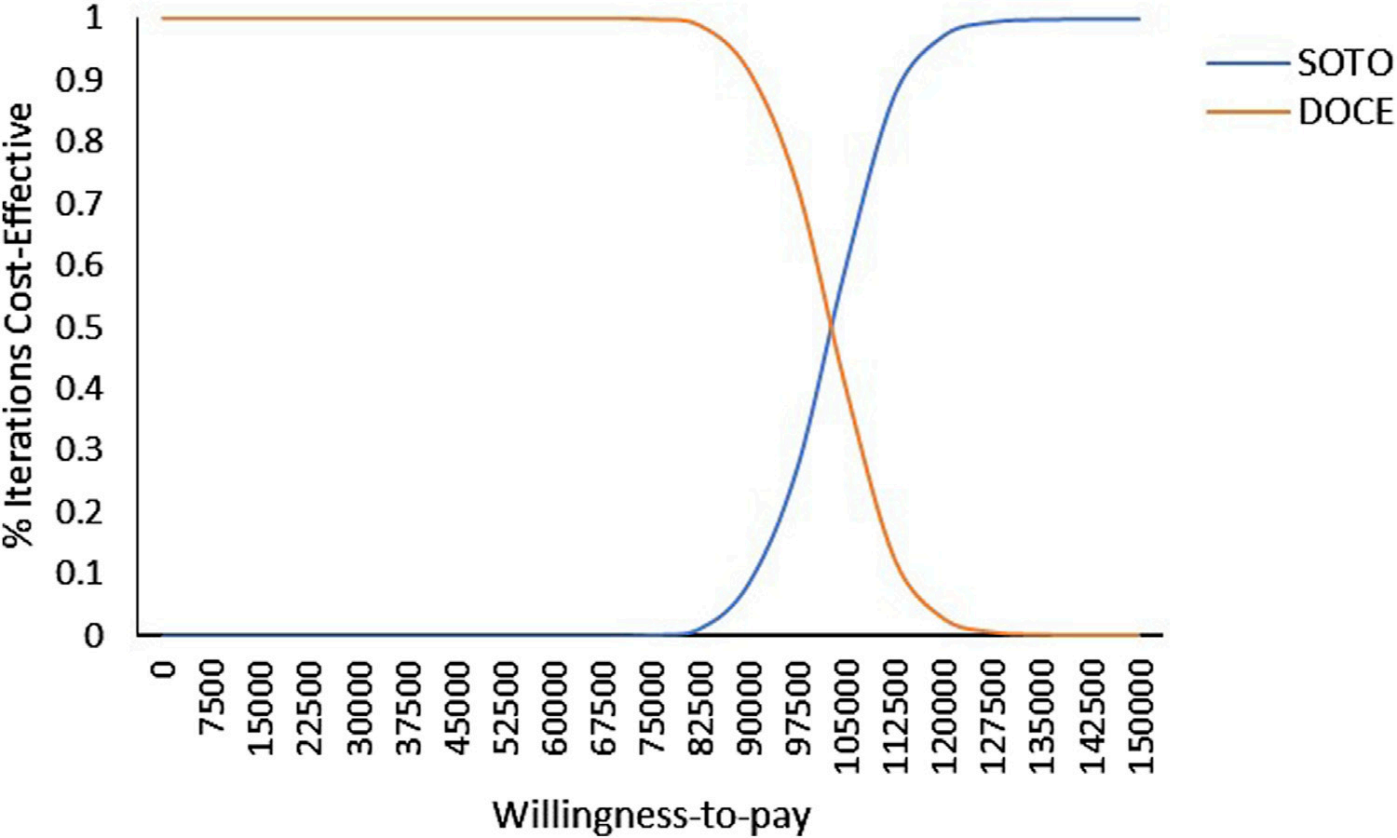
Cost-effectiveness acceptability curve of sotorasib and docetaxel taxel in Chinese perspective. Note: SOTO, sotorasib; DOCE, docetaxel.

As shown in Figure 4, within the range of WTP, docetaxel had the highest acceptable probability. With the increase of the value of WTP, the probability that docetaxel had an economic advantage gradually decreases. When the WTP exceed $102,500, the probability of sotorasib being cost-effective increases from 0% to 49% and gradually becomes higher than docetaxel. It can be seen that docetaxel had economic advantages in the range of the WTP threshold from the perspective of China.

**FIGURE 5:**
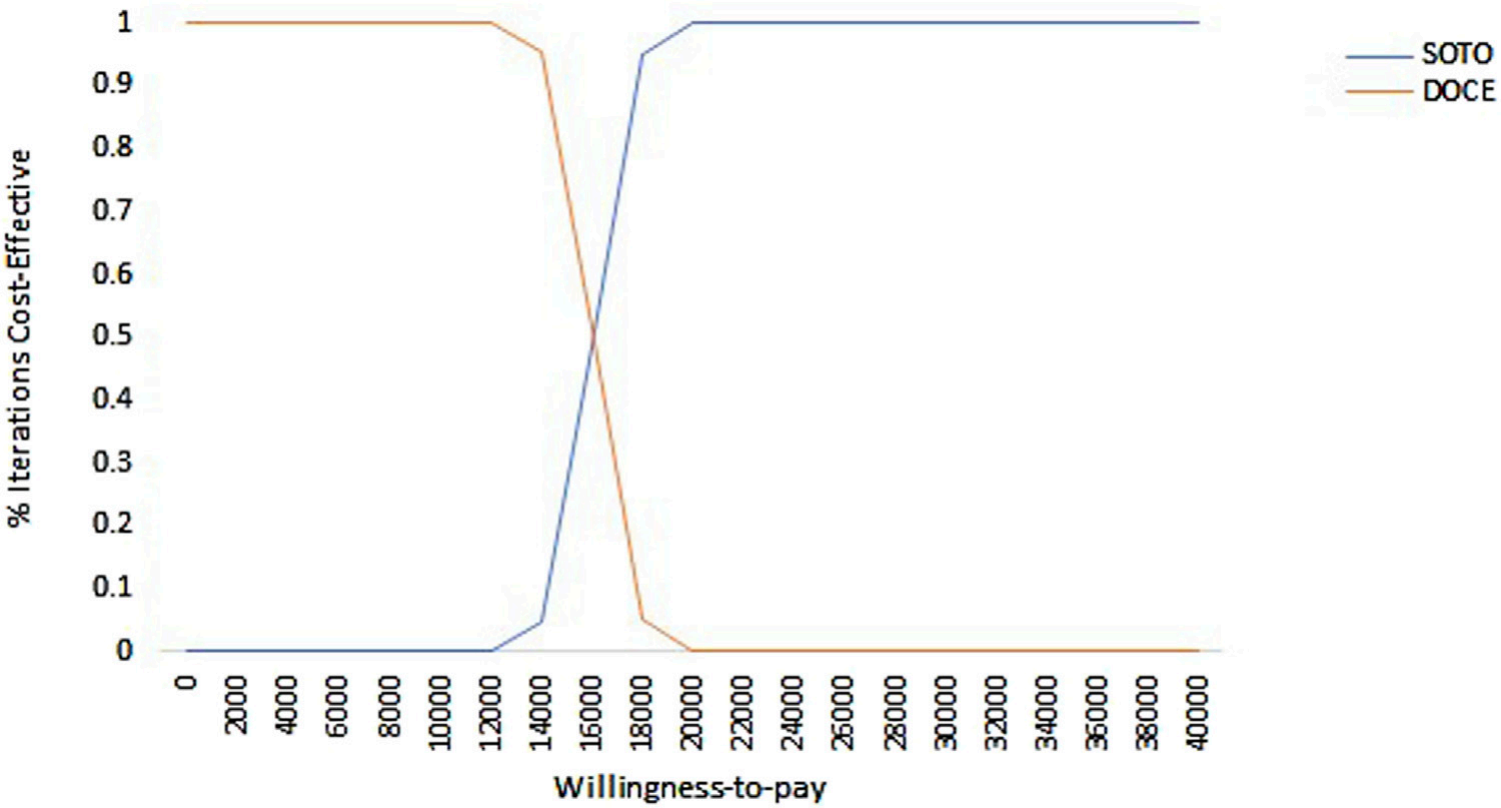
Cost-effectiveness acceptability curve of sotorasib and docetaxel in the US perspective. Note: SOTO, sotorasib; DOCE, docetaxel.

As shown in Figure 5, in the US, the acceptability probability of docetaxel was highest when the WTP value was less than $15,650. With the increase of WTP, the probability that sotorasib had a cost effect gradually increases. It can be seen that when the WTP was less than $15,650, docetaxel had economic benefits, and when the WTP was more than $15,650, sotorasib was more cost-effective.

## Discussion

Sotorasib was the first targeted drug approved by the FDA for the treatment of locally advanced or metastatic NSCLC with the KRAS^G12C^ mutation (Lu et al., 2023). However, sotorasib has not yet been listed in China, so it is urgent to evaluate the cost effectiveness of sotorasib. At the same time, it was necessary to comprehensively evaluate the cost effectiveness of sotorasib from multiple perspectives in China and the US. We hope that our research can provide reference for the listing of sotorasib in China.

The results of this study showed that sotorasib gained 0.09 QALY more than docetaxel, and ICER was $102701.84/QALY. In the US, sotorasib got 0.35 QALY more relative to docetaxel and ICER got $15,976.50/QALY. These results showed that sotorasib had not an economic advantage in China, but had an economic advantage in the US. The conclusion of univariate sensitivity analysis showed that the probability of sotorasib having economic benefits gradually increases when the cost of follow-up examination decreases in China. The probabilistic sensitivity analysis results showed that the acceptability of sotorasib gradually increases with the increase of WTP in China or the US, and sotorasib had cost effect when China’s WTP value increases to $102500, while the US only needs to increase to $15650 US to have cost effect.

One-way sensitivity analyses showed that the lower the cost of follow-up, the higher the economic benefit of sotorasib. The possible reason for this is that this study included the cost of examinations such as brain MRI, bone scan, and CT in the cost of follow-up. The PSA results suggest that an increase in WTP leads to a gradual increase in the acceptability of sotorasib, both in China and the United States. Nowadays, as the economic level of the world’s population continues to rise and the WTP increases each year, the acceptability of sotorasib will gradually rise above that of docetaxel to become an economically effective second-line treatment option.

A recent study evaluated the long-term efficacy of sotorasib in patients with advanced NSCLC with KRASG^12C^ mutation showed that a quarter achieved long-term benefits and almost no late-onset treaty-related toxicity, and sotorasib showed a good safety profile (Dy et al., 2023). At present, clinical trials of sotorasib were also being carried out in China, and CodeBreak105 (NCT4380753) study was a Phase I study to explore the efficacy of sotorasib monotherapy in Chinese patients with KRAS ^G12C^ mutation. Therefore, if the price of sotorasib can be reduced, it was of great significance for Chinese lung cancer patients with KRAS^G12C^ mutation to choose sotorasib treatment. The establishment of the collection system in China had greatly reduced the price of drugs and greatly reduced the economic burden of cancer patients (State Council of China, 2021).

This study have some highlights should be noted. First, sotorasib had been approved in several countries (US Food and Drug Administration, 2021; BioSpace, 2022; European Medicines Agency, 2022), and there was little data on its economic evaluation. Although sotorasib had not yet been approved in China, clinical trials had already been conducted in China. Second, We used the Markov model to analyze the cost effect of sotorasib from the perspectives of China and the US, which was helpful for our decision to choose treatment options.

This study also had the following limitations. Firstly, this study did not consider the utility value decline caused by adverse reactions. Secondly, due to the lack of health utility evaluation studies in Chinese patients, the extraction of relevant health utility values from published literature may cause a certain degree of bias to the conclusions. In the single factor sensitivity analysis in China, the variation of the utility value of PFS will affect the final conclusion. Second, most of the data comes from RCT studies, which differ from real-world data, so decisions should be made when applied to the clinical practice. Finally, the incidence of AEs was mainly ≥3, and other AE with low incidence but high cost were not considered. Univariate sensitivity analysis in the US showed that the cost of adverse events for treating diarrhea had an impact on the model.

## Conclusion

Our results showed that sotorasib had cost effect from the perspective of the US, and sotorasib had no cost effect from the perspective of China, and only when the WTP exceeds $102,500, the probability of sotorasib having cost effect increases from 0% to 49%.

## Data Availability

The original contributions presented in the study are included in the article/Supplementary Material, further inquiries can be directed to the corresponding author.
