# Supplementary material for: Cost-effectiveness of sotorasib as a second-line treatment for non-small cell lung cancer with KRASG12C mutation in China and the United States
Source: Front Pharmacol. 2024 Jun 14;15:1348688. doi: 10.3389/fphar.2024.1348688 (PMC11211580; doi:10.3389/fphar.2024.1348688)
Supplement: Supplementary file 1 [file DataSheet1.docx]

Supplementary Material


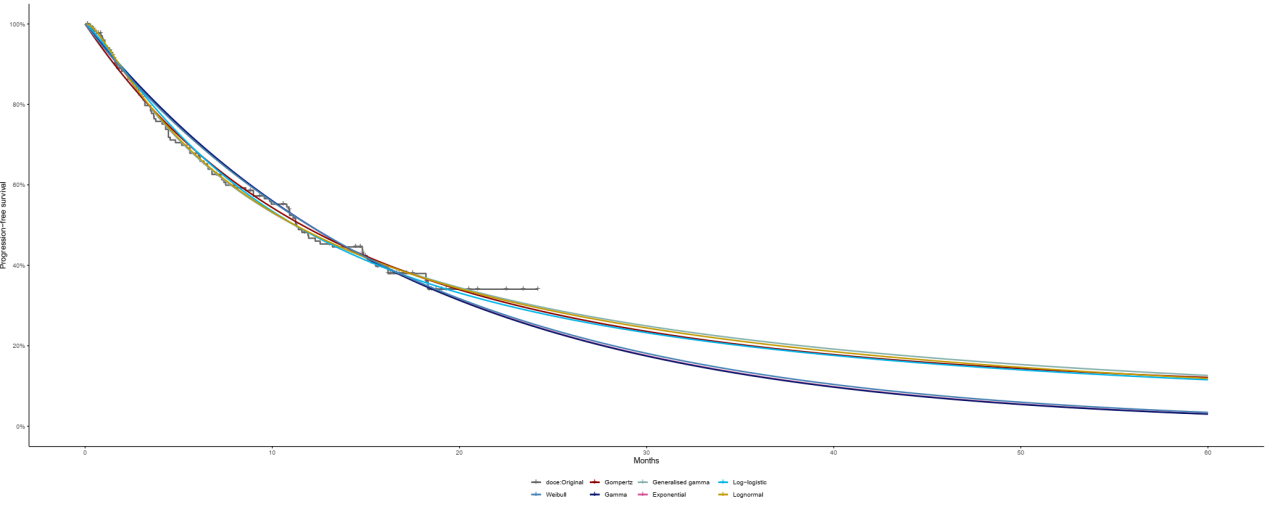


**Supplementary Figure1.** Comparison between reconstructed Kaplan-Meier curve for Progression Free Survival and all the parametric fitting curves among patients with docetaxel


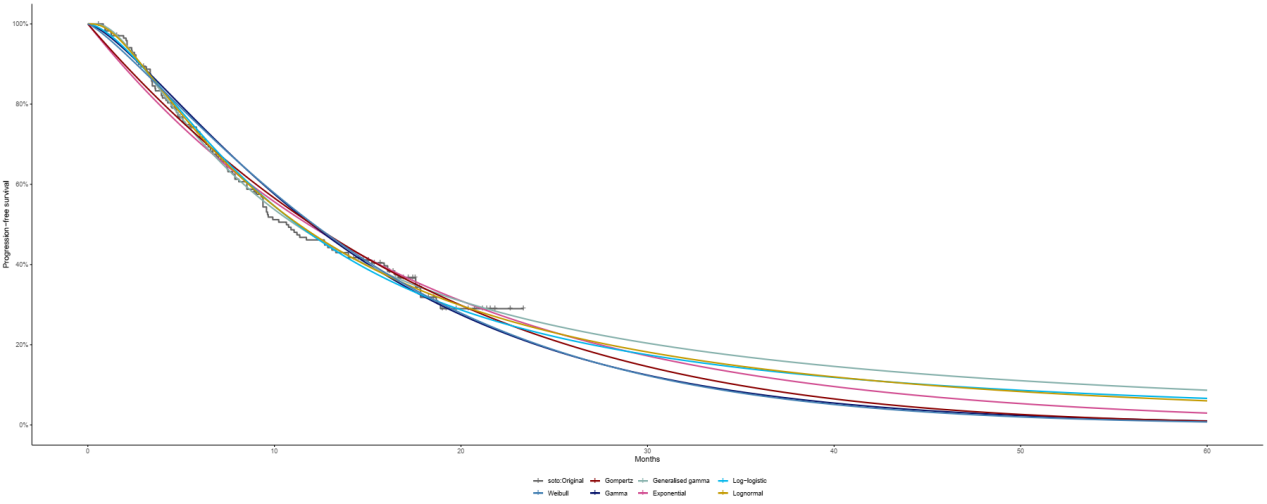


**Supplementary** **Figure2** Comparison between reconstructed Kaplan-Meier curve for Progression Free Survival and all the parametric fitting curves among patients with Sotorasib


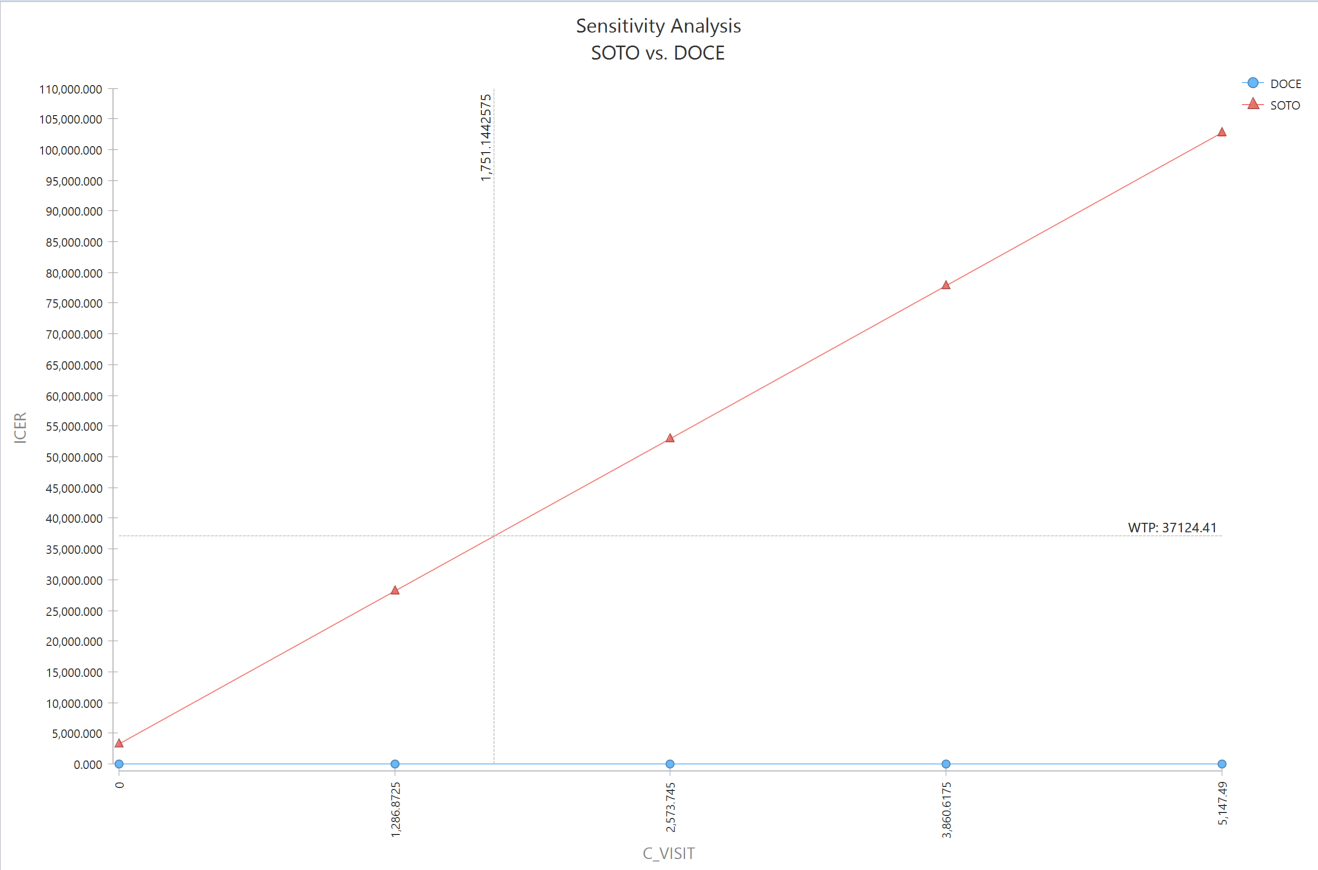


**Supplementary Figure3.** Unitary sensitivity analysis

| **Supplementar**y **Table1.** Parameters input to the model |  | **US perspective** | | | | |  |
| --- | --- | --- | --- | --- | --- | --- | --- |
| **Items** | **Drug** | **Variable Name** | **Parameter Values** | **upper limit** | **lower limit** | **Distribution** |  |
| **Incidence of adverse reactions** | Sotorasib | risk_Diarrhoea_S | 0.2 | 0.24 | 0.16 | Beta |  |
|  |  | risk_Fatigue_S | 0.01 | 0.012 | 0.008 | Beta |  |
|  |  | risk_Nausea_S | 0.02 | 0.024 | 0.016 | Beta |  |
|  |  | risk_Anaemia_S | 0.01 | 0.012 | 0.008 | Beta |  |
|  |  | risk_Decreasedappetite_S | 0.03 | 0.036 | 0.024 | Beta |  |
|  |  | risk_Asthenia_S | 0.01 | 0.012 | 0.008 | Beta |  |
|  |  | risk_ALT_S | 0.13 | 0.156 | 0.104 | Beta |  |
|  |  | risk_AST_S | 0.09 | 0.108 | 0.072 | Beta |  |
|  | Docetaxel | risk_Diarrhoea_D | 0.03 | 0.036 | 0.024 | Beta |  |
|  |  | risk_Fatigue_D | 0.09 | 0.108 | 0.072 | Beta |  |
|  |  | risk_Nausea_D | 0.01 | 0.012 | 0.008 | Beta |  |
|  |  | risk_Anaemia_D | 0.05 | 0.06 | 0.04 | Beta |  |
|  |  | risk_Stomatitis_D | 0.02 | 0.024 | 0.016 | Beta |  |
|  |  | risk_Asthenia_D | 0.04 | 0.048 | 0.032 | Beta |  |
|  |  | risk_Neutropenia_D | 0.18 | 0.216 | 0.144 | Beta |  |
|  |  | risk_Neuropathy peripheral_D | 0.01 | 0.012 | 0.008 | Beta |  |
|  |  | risk_Oedema peripheral_D | 0.01 | 0.012 | 0.008 | Beta |  |
|  |  | risk_Myalgia_D | 0.02 | 0.024 | 0.016 | Beta |  |
|  |  | risk_Arthralgia_D | 0.01 | 0.012 | 0.008 | Beta |  |
|  |  | risk_Mucositis_D | 0.02 | 0.024 | 0.016 | Beta |  |
|  |  | risk_Malaise_D | 0.01 | 0.012 | 0.008 | Beta |  |
|  |  | risk_Febrile neutropenia_D | 0.08 | 0.096 | 0.064 | Beta |  |
|  |  | risk_Pneumonia_D | 0.05 | 0.06 | 0.04 | Beta |  |
| **The cost of follow-up** **($)** |  | C_consultation_fee | 68.04 | 81.648 | 54.432 | Beta |  |
|  |  | C_CT | 425.280 | 510.336 | 340.224 | Beta |  |
|  |  | C_MRI | 90.480 | 109.800 | 71.160 | Beta |  |
|  |  | C_BS | 40.330 | 48.396 | 32.264 | Beta |  |
|  |  | C_CBC | 7.770 | 9.324 | 6.216 | Beta |  |
|  |  | C_BIOTH | 512.08 | 614.496 | 409.664 | Beta |  |
|  |  | C_REPET | 114.540 | 137.448 | 91.632 | Beta |  |
|  |  | C_REMRI | 90.480 | 109.800 | 71.160 | Beta |  |
|  |  | C_GENE | 1943.210 | 2331.852 | 1554.568 | Beta |  |
| **The cost of hospitalization ($)** |  | C_bed | 1715.00 | 2058.00 | 1372.00 | Beta |  |
|  |  | C_cwere | 299.90 | 359.88 | 239.92 | Beta |  |
|  |  | C_hospitalization examination | 772.23 | 926.68 | 617.78 | Beta |  |
|  |  | C_trans | 144.39 | 173.27 | 115.51 | Beta |  |
|  |  | **Chinese perspective** | | | | |  |
| **Items** |  | **Variable Name** | **Parameter Values** | **upper limit** | **lower limit** | **Distribution** |  |
| **Incidence of adverse reactions** | **sotorasib** | risk_Diarrhoea_S | 0.2 | 0.24 | 0.16 | Beta |  |
|  |  | risk_Fatigue_S | 0.01 | 0.012 | 0.008 | Beta |  |
|  |  | risk_Nausea_S | 0.02 | 0.024 | 0.016 | Beta |  |
|  |  | risk_Anaemia_S | 0.01 | 0.012 | 0.008 | Beta |  |
|  |  | risk_Decreased appetite_S | 0.03 | 0.036 | 0.024 | Beta |  |
|  |  | risk_Asthenia_S | 0.01 | 0.012 | 0.008 | Beta |  |
|  |  | risk_ALT_S | 0.13 | 0.156 | 0.104 | Beta |  |
|  |  | risk_AST_S | 0.09 | 0.108 | 0.072 | Beta |  |
|  | **docetaxel** | risk_Diarrhoea_D | 0.03 | 0.036 | 0.024 | Beta |  |
|  |  | risk_Fatigue_D | 0.09 | 0.108 | 0.072 | Beta |  |
|  |  | risk_Nausea_D | 0.01 | 0.012 | 0.008 | Beta |  |
|  |  | risk_Anaemia_D | 0.05 | 0.06 | 0.04 | Beta |  |
|  |  | risk_Stomatitis_D | 0.02 | 0.024 | 0.016 | Beta |  |
|  |  | risk_Asthenia_D | 0.04 | 0.048 | 0.032 | Beta |  |
|  |  | risk_Neutropenia_D | 0.18 | 0.216 | 0.144 | Beta |  |
|  |  | risk_Neuropathy peripheral_D | 0.01 | 0.012 | 0.008 | Beta |  |
|  |  | risk_Oedema peripheral_D | 0.01 | 0.012 | 0.008 | Beta |  |
|  |  | risk_Myalgia_D | 0.02 | 0.024 | 0.016 | Beta |  |
|  |  | risk_Arthralgia_D | 0.01 | 0.012 | 0.008 | Beta |  |
|  |  | risk_Mucositis_D | 0.02 | 0.024 | 0.016 | Beta |  |
|  |  | risk_Malaise_D | 0.01 | 0.012 | 0.008 | Beta |  |
|  |  | risk_Febrile neutropenia_D | 0.08 | 0.096 | 0.064 | Beta |  |
|  |  | risk_Pneumonia_D | 0.05 | 0.06 | 0.04 | Beta |  |
| **The cost of**  **Follow-up ($)** |  | C_consultation_fee | 154 | 300 | 8 | Beta |  |
|  |  | C_CT | 782.75 | 872.02 | 693.49 | Beta |  |
|  |  | C_MRI | 199.97 | 221.63 | 199.97 | Beta |  |
|  |  | C_BS | 99.64 | 0.00 | 0.00 | Beta |  |
|  |  | C_BATH | 72.92 | 204.76 | 72.92 | Beta |  |
|  |  | C_CBC | 2.60 | 0.00 | 0.00 | Beta |  |
|  |  | C_BIOTH | 56.32 | 0.00 | 0.00 | Beta |  |
|  |  | C_REPET | 1400.68 | 0.00 | 0.00 | Beta |  |
|  |  | C_REMRI | 199.97 | 221.63 | 199.97 | Beta |  |
|  |  | C_GENE | 2310.40 | 2888.00 | 1732.80 | Beta |  |
| **The cost of**  **hospitalization ($)** |  | C_bed | 14.58 | 25.99 | 3.18 | Beta |  |
|  |  | C_cwere | 3.47 | 4.04 | 2.89 | Beta |  |
|  |  | C_hospitalization examination | 4.33 | 0.00 | 0.00 | Beta |  |
|  |  | C_trans | 1.73 | 0.00 | 0.00 | Beta |  |
|  |  | C_prepweretion | 2.17 | 0.00 | 0.00 | Beta |  |
